# Digital Hammering Inspection for Social Infrastructure

**DOI:** 10.3390/s26216805

**Published:** 2026-10-28

**Authors:** Yoshihiro Isobe, Takashi Matsunaga, Ryota Ogawa, Mitsuyuki Sagisaka, Hiroaki Fujiyoshi, Motomu Ishii, Jun Tomiyama, Tomonori Yamada

**Affiliations:** 1 Engineering Service Department, Nuclear Fuel Industries, Ltd., Kumatori-cho 590-0481, Osaka, Japan; takashi.matsunaga@cresco.co.jp (T.M.); ryota.ogawa.t83@mail.toshiba (R.O.); mitsuyuki.sagisaka.x59@mail.toshiba (M.S.); motomu.ishii.d61@mail.toshiba (M.I.); 2 Civil Engineering Program, School of Engineering, University of the Ryukyus, Nishihara-cho 903-0213, Okinawa, Japan; jun-t@tec.u-ryukyu.ac.jp; 3 Department of Systems Innovation, School of Engineering, The University of Tokyo, Tokyo 113-8656, Japan; tyamada@sys.t.u-tokyo.ac.jp

**Keywords:** acoustic emission sensor, quality management, age-related management, digital hammering inspection, non-destructive testing, infrastructure

## Abstract

Although conventional hammering inspection remains a standard practice across various industries, its effectiveness is often limited by a heavy reliance on the inspector’s subjective skill, leading to qualitative and inconsistent results. To address these limitations, this study introduces a digital hammering inspection system integrated with Acoustic Emission (AE) sensors, designed for enhanced quality control and long-term aging management. The system has been extensively implemented in the diagnostic evaluation of social infrastructure, encompassing both metallic and concrete structures, including various anchor systems, guardrail supports, and concrete bridge components. By leveraging the principles of natural vibration, the system demonstrates the potential to provide an objective, quantitative, and recordable screening process that may help reduce human error and facilitate high-speed data acquisition. Furthermore, its lightweight design and portability may facilitate field application, enabling on-site diagnostics with the potential to be applied across diverse engineering environments.

## 1. Introduction

**Figure 1:**
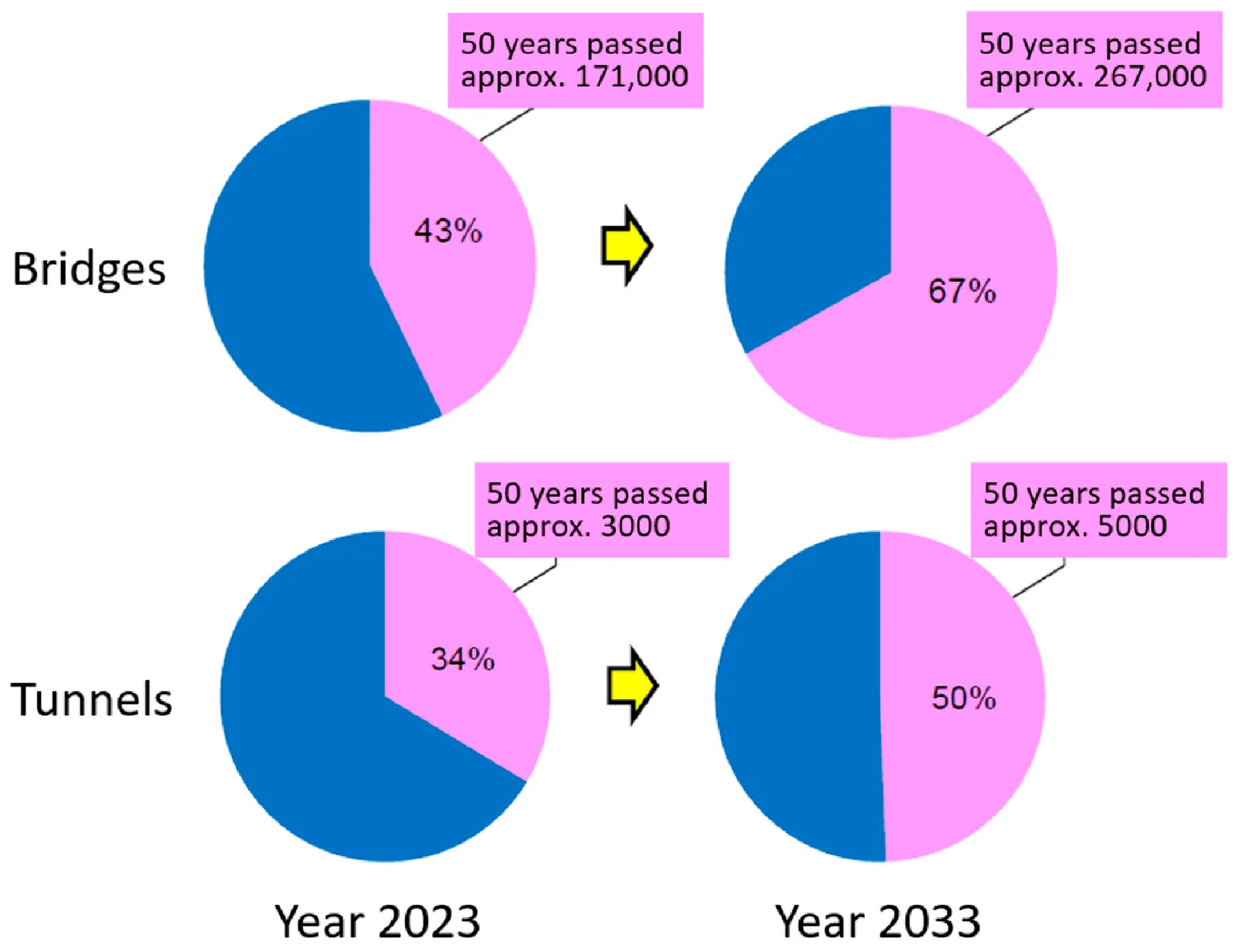
Aging bridges and tunnels in Japan that have been in service for more than 50 years.

Social infrastructure in Japan is facing significant aging, and the number of road sections that have been in service for more than 50 years continues to increase, as shown in Figure 1 [1]. As these structures age, the deterioration of mechanisms such as fatigue, corrosion, and material degradation become more pronounced, making systematic maintenance increasingly important. Maintenance of road structures—including bridges, tunnels, and auxiliary facilities—has therefore become an urgent issue, and the importance of ensuring their soundness through periodic and efficient inspections has become even more essential for maintaining safety and reliability. In particular, adequate repairs implemented on the basis of early inspections offer the advantage of reducing the total lifecycle cost of social infrastructure during the in-service period, as they help prevent minor defects from developing into severe and costly damage.

Although close visual inspections and conventional hammering inspections have long been employed in fields such as civil engineering, public transportation, construction, and power generation, these traditional methods exhibit inherent limitations. Because they rely heavily on the skill, experience, and intuition of individual inspectors, the resulting on-site judgments tend to be subjective and largely qualitative, leading to variability in diagnostic accuracy. Moreover, as infrastructure systems continue to age and the demand for more reliable condition assessment increases, the dependence on such inspector-dependent techniques poses challenges in ensuring consistency, reproducibility, and traceability of inspection results across different sites and personnel.

Therefore, a large number of digital hammering inspection systems incorporating sensors have been proposed, and several of them have been standardized, particularly for concrete structures such as bridges and tunnels [2,3,4,5,6,7,8,9,10,11,12,13,14,15]. According to previous studies, conventional hammering inspection has predominantly relied on subjective assessments conducted by human inspectors. To address this limitation, various sensor-based digital hammering inspection methods have been proposed. However, there are still only limited examples of these digital hammering inspection techniques being applied in the field to a wide variety of road infrastructure structures. While the impact-echo method is recognized as a highly valuable non-destructive evaluation technique and has been extensively applied to concrete structures, its application to other types of structures remains relatively limited. The key feature of this study is the development of a method that enables a seamless transition from conventional hammering inspections by using the same inspection hammer while recording the impact response with AE sensors. The proposed method was applied not only to concrete structures but also to a variety of metallic road infrastructure structures.

We have developed a digital hammering inspection system equipped with an AE sensor (hereinafter referred to as the AE digital hammering inspection system) and have applied it to a wide range of social infrastructure fields, leveraging the fundamental principles of natural vibration phenomena. With the AE digital hammering inspection system, the sensor is placed on the inspection surface with one hand, without any need for pretreatment such as flattening or polishing, and the area adjacent to the sensor is subsequently struck with a conventional hammer. Since the battery-powered system is portable and easy to deploy in the field, it enables simplified on-site diagnosis, and when necessary, more detailed analysis can subsequently be performed in the office based on the recorded inspection data. This provides a rapid non-destructive screening method for assessing the integrity of social infrastructure prior to conducting detailed, costly, and time-consuming state-of-the-art non-destructive testing (NDT) procedures.

The natural frequency measured by digital hammering inspection is primarily governed by the mass, geometry, and stiffness of the inspected object, as well as by the boundary constraints imposed by its surrounding environment. When improper installation, structural defects, or aging-related deterioration are present, the peak frequency deviates from the reference value characteristic of a sound condition.

This paper presents the fundamental principles of digital hammer inspection, which evaluates structural conditions based on the natural frequency characteristics of objects, and reports the results of applying the AE digital hammering system to a variety of road-related metal components and concrete structures. Through these applications, the study suggests the system’s broad applicability to the diagnosis of social infrastructure for both quality management and age-related deterioration management.

## 2. Materials and Methods

A portable AE-based digital hammering inspection system, together with the fundamental principles of natural vibration phenomena that underpin its application to actual social infrastructure, is summarized below.

### 2.1. Portable AE Digital Hammering Field Inspection System

**Figure 2:**
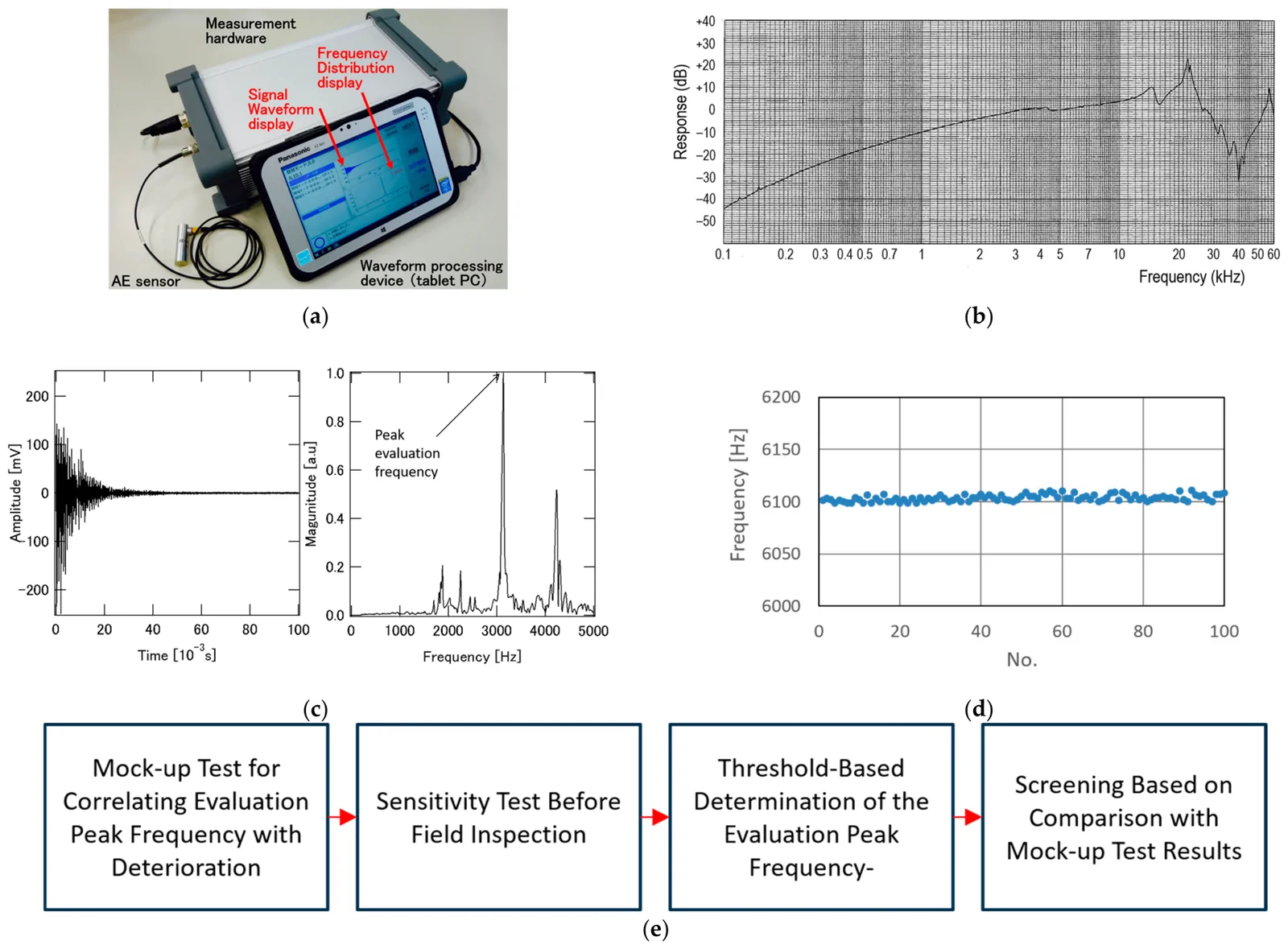
AE digital hammering inspection system and an example of signal analysis. (**a**) Portable AE-based digital hammering field inspection system. (**b**) Frequency response in the 1000–10,000 Hz range. (**c**) Signal analysis using FFT (Fast Fourier Transform) processing. (**d**) Results of the natural frequency obtained from 100 consecutive sensitivity tests. (**e**) Inspection flowchart.

When using the portable AE-based digital hammering field inspection system (Figure 2a), the inspection target is struck with a conventional hammer to induce vibration, while the sensor is placed near the impact point with one hand, without any need for surface pretreatment such as flattening or polishing. The entire procedure requires only a few seconds per inspection point. The frequency response of the broad-band AE sensor (AE-900S-WB, NF Corporation, Yokohama, Japan) is shown in Figure 2b. The frequency response in the 1000–10,000 Hz range, which is primarily used for diagnosing social infrastructure, is nearly flat; therefore, the sensor is well-suited for stable vibration measurement. The natural vibration peak is extracted from the frequency spectrum of the measured waveform after FFT (Fast Fourier Transform) processing, and the frequency is used as an evaluation index (Figure 2c). The sampling rate during the measurement was 250 kHz, and the acquisition time was 0.1 s, resulting in a frequency resolution of 10 Hz. For FFT processing, the 0.1-s waveform was zero-padded on both sides and transformed as a 1-s data record. As a result, an apparent frequency resolution of 1 Hz was achieved, although the actual frequency resolution remained 10 Hz. This allowed the peak frequency to be identified at 1 Hz intervals.

Prior to field inspection, a sensitivity test was performed using a SUS304 cold-drawn stainless-steel rod (diameter: 15 mm, length: 400 mm). The theoretical natural frequency of its first longitudinal vibration mode is 6119 Hz, and field inspections are conducted only after confirming that the measured frequency was within ±50 Hz of the theoretical value, corresponding to an error of less than 1.0%. Furthermore, the standard deviation (1σ) is typically around 3 Hz, corresponding to a variation of less than 1% (Figure 2d). Even in field inspections of actual structures, repeated measurements at the same location generally exhibit variations of a few percent.

The index frequency is determined by identifying natural vibration peaks that exceed a predefined threshold and selecting the lowest-frequency peak among them. The inspection flowchart is shown in Figure 2e. The predefined threshold has been empirically set to 0.3 based on our previous experimental results for both concrete and metal components. This value was selected because it allows the peak height of the peak evaluation frequency to be separated relatively easily from environmental noise. Typically, approximately three hammer impacts are applied at the same location, and these impacts can be completed within a few seconds including the real-time display of the frequency spectrum of the measured waveform on the tablet PC. In cases of elevated environmental noise, additional impacts are repeated as necessary, and the peak adopted for evaluation is determined based on engineering judgment using the signals obtained from the repeated measurements. The rationale for selecting the lowest-frequency natural vibration peak that exceeds the threshold is that this frequency corresponds to a lower-order vibration mode—typically a simple bending mode—that is not affected by overlapping peaks associated with different vibration modes or higher-order modes that appear in the higher-frequency range.

Using the predefined threshold, the evaluation peak frequency obtained from repeated hammer impacts at the same location generally remains within a variation of only a few percent. Furthermore, regarding the contact between the sensor and the structural surface, one of the advantages of the proposed inspection technique is that anyone can obtain comparable results after approximately half a day of practice. To ensure good contact conditions between the AE sensor and the structure, a silicone rubber sheet of approximately 1 mm thickness is attached to the front surface of the sensor.

**Figure 3:**
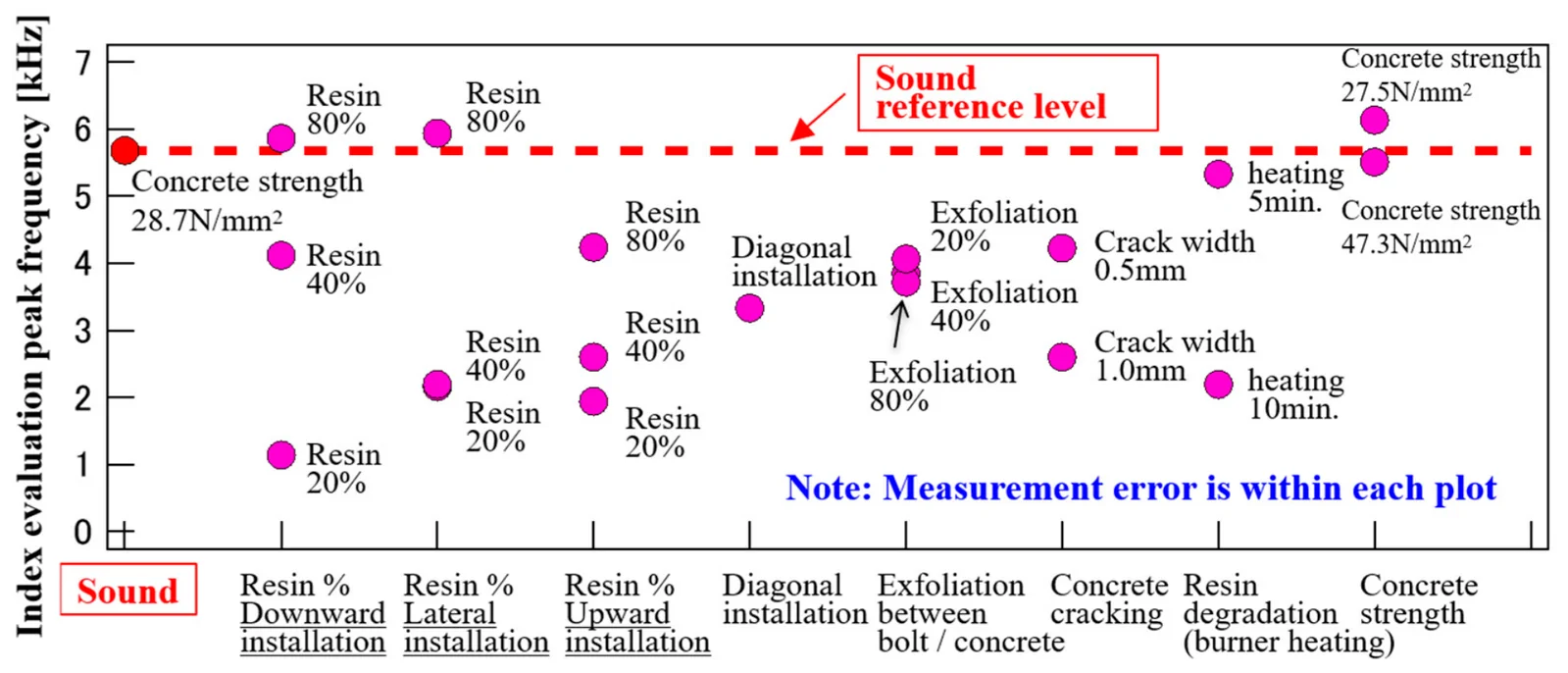
Relationship between construction defects, age-related deterioration, and evaluation peak frequency of M16 anchors.

The principle of natural vibration of a simple harmonic motion of spring is shown by Equation (1):

(1) $$f=\frac{1}{2\pi }\sqrt{\frac{k}{m}}$$

where *f* [Hz] is natural frequency, *k* [N/m] is spring constant and *m* [kg] is mass [16]. Equation (1) suggests how parameters such as mass and stiffness influence the natural frequency, as described below. The natural frequency increases when the mass or structural configuration becomes smaller, and when the stiffness or boundary restraint from the surrounding environment becomes greater. Considering these effects, an example of diagnosing post-installed adhesive M16 anchors based on mock-up tests is shown in Figure 3. When improper installation or age-related deterioration is present, the evaluated peak frequency generally decreases from the predetermined reference value corresponding to a sound condition. Specifically, when the amount of resin used for bonding is insufficient, when the anchor is installed diagonally, when debonding occurs between the bolt and the concrete, when cracks develop in the concrete, or when the resin deteriorates with age, the anchorage performance between the anchor and the concrete is weakened. As a result, the evaluated peak frequency decreases.

### 2.2. Digital Hammering Inspection Applied to Actual Social Infrastructure

To provide a comprehensive understanding of the methodology, the fundamental physical principles governing digital hammering inspection, particularly its practical implementation in the structural health monitoring of critical social infrastructure, are summarized below.

Equation (2) [16] can be applied to inspections of beam-shaped metal components, such as bolts and pipes, for which the fundamental vibration characteristics are governed primarily by their geometric dimensions and material properties.

(2) $$f_{n}=\frac{\lambda_{n}^{2}}{2\pi l^{2}}\;\sqrt{\frac{\mathrm{EI}}{\rho A}}$$

where *f* [Hz] is the natural frequency of bending, *n* is the mode order of vibration system, *A* [m^2^] is the cross section of beam, *I* [m^4^] is the second moment of area, *E* [N/m^2^] is the longitudinal elastic modulus, *λ* is the constant of constraint condition of beam, *ρ* [kg/m^3^] is the mass density and *l* [m] is the length of beam. Because Equation (2) is formulated for idealized, complete beam members, the actual geometry of components—such as the combined configuration of bolts and nuts—must be taken into account when applying it in practice. Moreover, since Equation (2) does not include a stress term, the axial tension force acting on bolts must also be considered when evaluating their condition.

For the inspection of plate-shaped concrete structures—such as bridge girders, deck slabs, and tunnel-lining concrete—alternative natural-frequency formulations appropriate for plate-type vibration behavior can be applied. From the frequency spectrum obtained through digital hammering inspection of concrete surfaces, attention is directed to the natural frequencies associated with longitudinal vibration and flexural vibration. The longitudinal vibration component is utilized for evaluating the web thickness of concrete members, whereas the flexural vibration component arises in shallow concrete layers and is indicative of defects such as voids or spalling. The natural frequency associated with longitudinal vibration is given by Equation (3). The flexural vibration component is described by Equation (4) for cases in which the concrete cover can be approximated as a rectangular plate and the surrounding boundary condition is assumed to be simply supported [16].

(3) $$f=\frac{V}{2D}=\frac{1}{2D}\sqrt{\frac{E}{\rho }\cdot \frac{1-\nu }{\left(1+\nu \right)\cdot \left(1-2\nu \right)}}$$

(4) $$f=\frac{\pi }{2}\sqrt{\frac{Eh^{2}}{12\rho (1-\nu^{2})}}\left[{\left(\frac{m}{a}\right)}^{2}+{\left(\frac{n}{b}\right)}^{2}\right]$$

*f*: natural frequency [Hz], *E*: longitudinal elastic modulus [Pa], *ρ*: mass density [kg/m^3^], *V*: velocity of elastic wave [m/s], *D*: web thickness [m], *a*, *b*: rectangular plate size, *h*: thickness of exfoliation region [m], *ν*: Poisson’s ratio, *m*, *n*: order of vibration mode.

These natural frequencies deviate from those of a sound condition when the apparent longitudinal elastic modulus decreases along the web-thickness direction due to cracking, internal voids, or reductions in compressive strength, or when flexural vibration of the concrete cover occurs at the surface.

## 3. Results and Discussion

Examples of on-site digital hammering inspections, conducted in accordance with the fundamental principles represented by Equations (2)–(4) for both metal components and concrete structures, are summarized below.

### 3.1. Post-Installed Expansion Anchors

**Figure 4:**
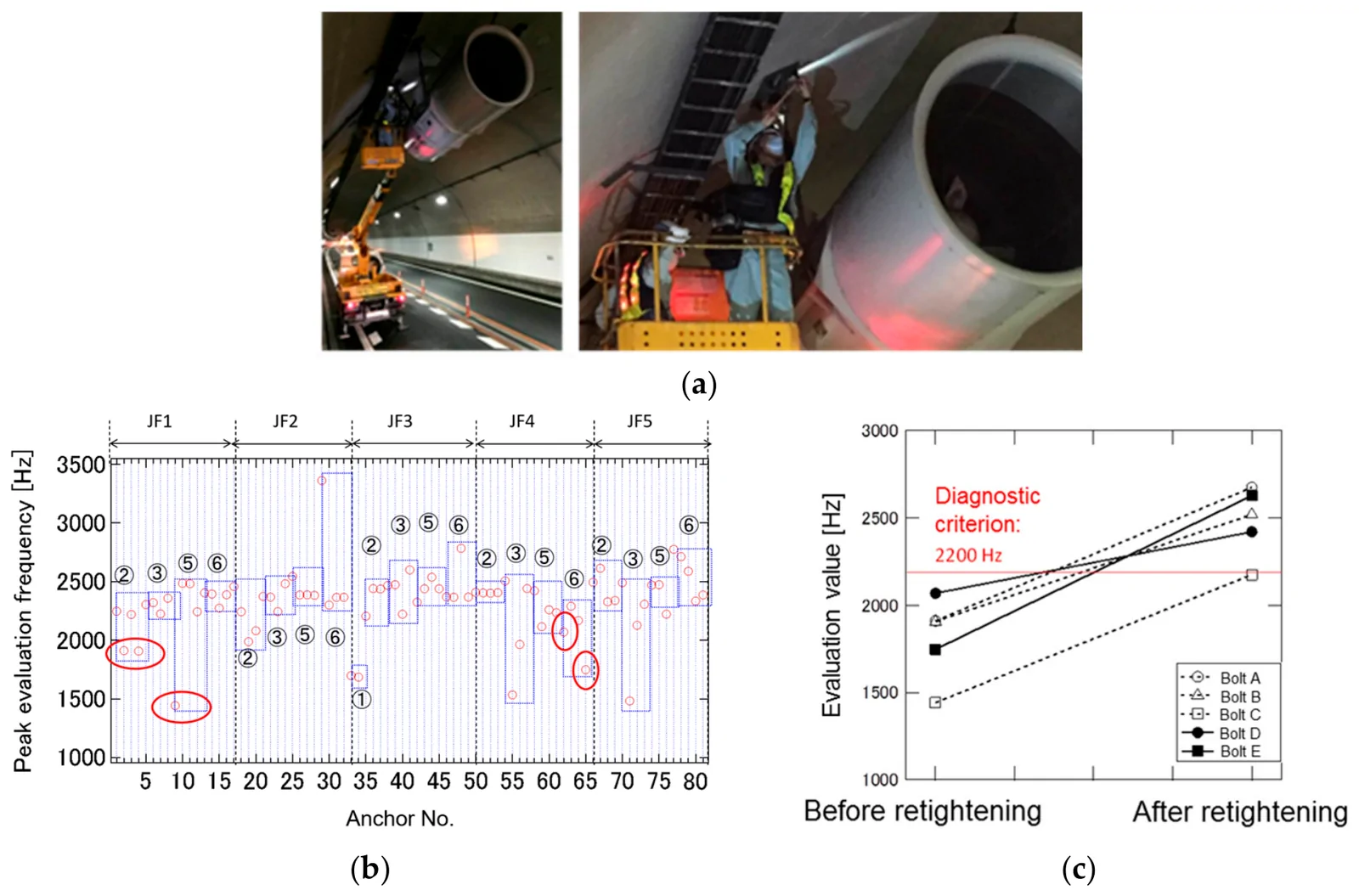
Examples of on-site inspection of jet-fan anchors. (**a**) On-site inspection of jet fans. (**b**) Inspection results of anchors supporting jet fans (**c**) Before and after retightening anchors.

On-site inspection results for post-installed expansion M24 anchors supporting five jet fans (JFs) in an expressway tunnel in Figure 4a are presented below. The circled numbers 2, 3, 5, and 6 in Figure 4b denote the four pieces of metal hardware used to suspend each JF by chains, with four anchors installed on each hardware unit. Based on previous mock-up tests that established the relationship between anchor-tightening torque and evaluation peak frequency, the reference value for the evaluation peak frequency under sound conditions was set to above 2200 Hz, corresponding to the specified anchor-tightening torque of 240 N·m. In the mock-up test, four M24 anchors were embedded in a 1 m × 1 m × 1 m concrete block using the actual mounting hardware employed to secure JFs to tunnel-lining concrete. To simulate the load of a JF, a concrete block weighing more than 1 ton was suspended from the center of the mounting hardware, and the evaluation peak frequency was evaluated by the AE-based digital hammering inspection.

Five JF anchors with evaluation peak frequencies below the reference value (Anchor Nos. 2, 4, 9, 62, and 65, shown from left to right by red circles in Figure 4b) were selected for torque verification using a torque wrench. All five anchors exhibited tightening torque values lower than the design specification of 240 Nm. Note that the immediate loss of jet-fan safety is unlikely even when several anchors exhibit reduced tightening torque, because each jet fan is supported by a total of sixteen anchors. After retightening the anchors to 240 Nm, the evaluation peak frequencies shifted to higher values, reflecting the increased constraint introduced by the retightening, as shown in Figure 4c.

### 3.2. Foundation Bolts

**Figure 5:**
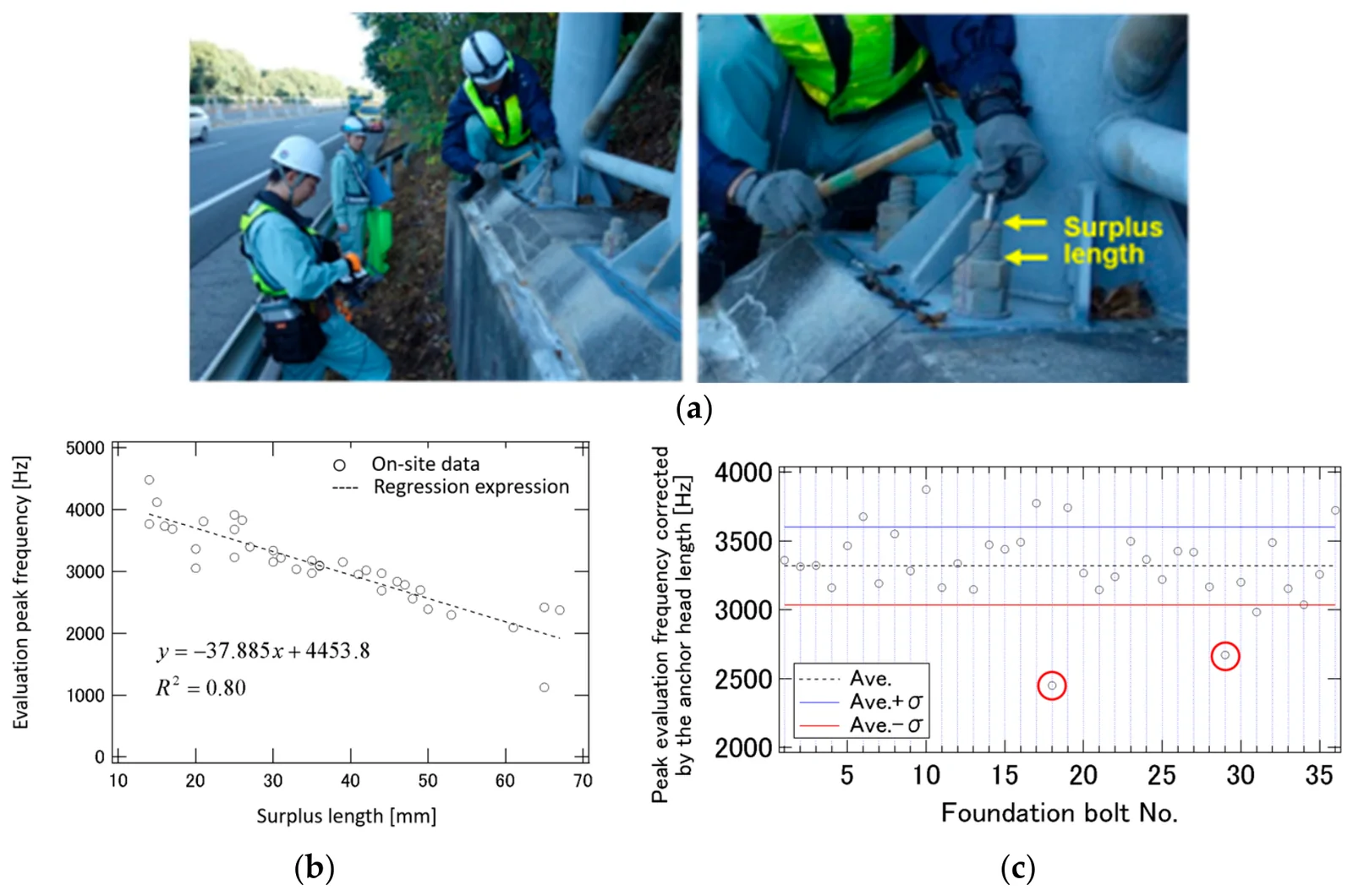
(**a**) Inspection for foundation bolts of expressway sign structures. (**b**) Evaluation peak frequencies of thirty-six sound M36 foundation bolts as a function of surplus length, together with a first-order regression line. (**c**) Evaluation peak frequencies obtained from the inspection results and corrected using the first-order regression line.

Inspection results for the foundation bolts of expressway sign structures shown in Figure 5a are presented below. In routine practice, visual inspection is first performed to identify signs of corrosion before conducting ultrasonic testing [17]. Figure 5b shows the evaluation peak frequencies of thirty-six sound M36 foundation bolts, sorted by their surplus length—that is, the protruding length above the nut. The results indicate a clear tendency for the evaluation peak frequency to shift toward lower values as the surplus length increases. This trend is consistent with Equation (2), which predicts a decrease in natural frequency as the effective vibration length becomes longer. Accordingly, deterioration-induced changes in the natural frequency of foundation bolts can be evaluated by correcting for the influence of surplus length.

For the M36 foundation bolt, a first-order regression line was derived, and the correction was performed using a gradient of −37.9 Hz/mm. Figure 5c presents the inspection results for the foundation bolts of expressway signs after applying the frequency-correction gradient of −37.9 Hz/mm, using a surplus bolt length of 30 mm as the reference condition. The regression-corrected evaluation peak frequencies of the foundation bolts averaged approximately 3318 Hz, with a standard deviation of about 282 Hz. In addition, two bolts exhibited markedly lower frequencies, falling well beyond one standard deviation on the low-frequency side, which is attributable to reduced constraint conditions caused by factors such as insufficient tightening torque, bolt corrosion, or deterioration of the concrete foundation.

The inspection requires approximately 90 s per bolt, including the measurement of the surplus length, and does not involve any surface preparation, a feature that is particularly advantageous for field applicability.

### 3.3. Ground Anchors

**Figure 6:**
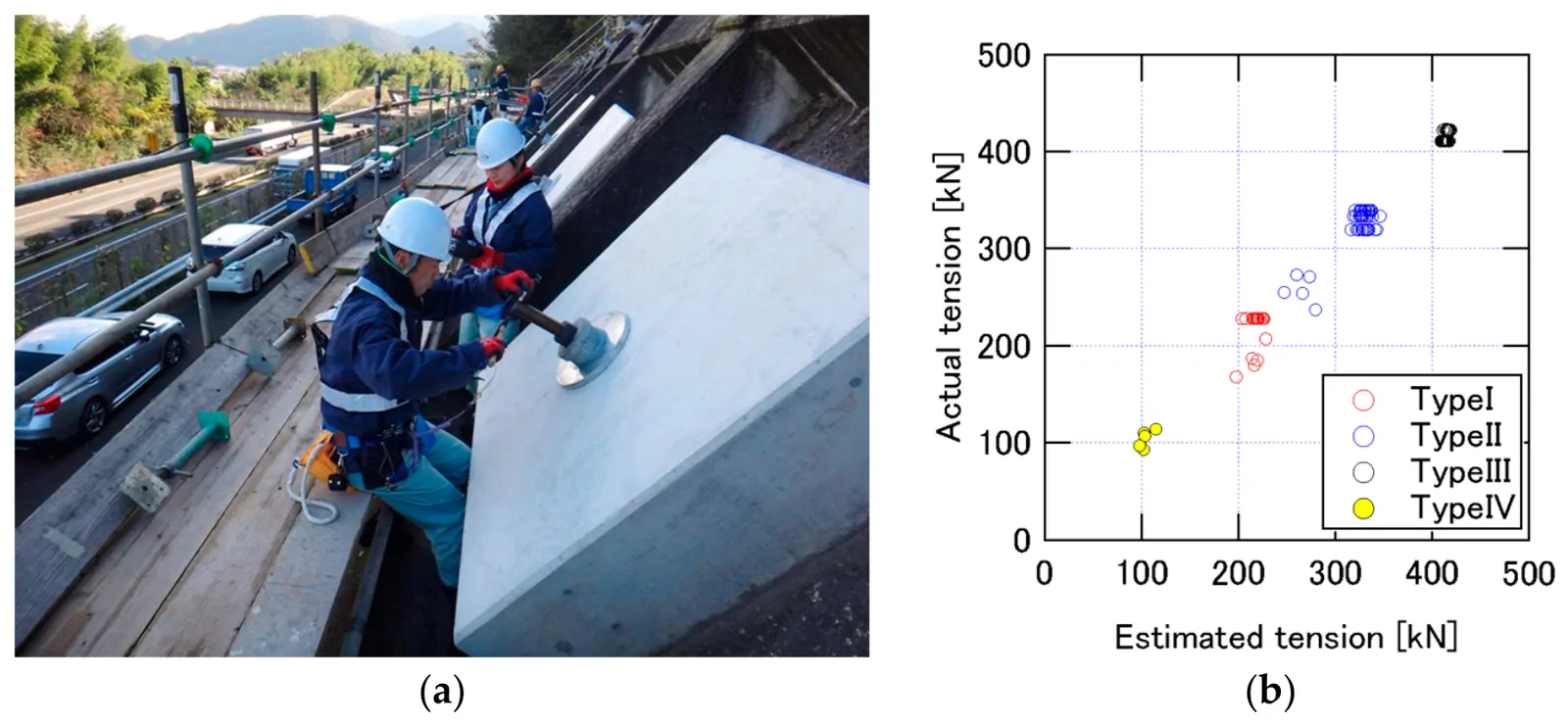
Field inspection of residual tensile force of ground anchors. (**a**) Ground anchors. (**b**) Comparison of actual tensile forces obtained from the lift-off tests with the tensile forces estimated by the inspection.

Ground anchors are employed to stabilize cut slopes, as illustrated in Figure 6a. The residual tensile force of these anchors has traditionally been measured using lift-off testing [18]; however, the development of a simpler alternative method for tension assessment is needed to reduce maintenance time and cost.

Because Equation (2) does not include a stress variable for the bending vibration of a beam, the residual tensile force must be explicitly considered when analyzing the bending vibration of ground anchors. Accordingly, a multiple-regression approach incorporating tensile force was adopted, as expressed in Equation (5).

(5) $$S=\alpha \cdot f+\beta /l^{2}+\gamma$$

where *S* is estimated tensile force (kN), *f* is evaluation peak frequency (Hz), *l* is surplus length, and *α*, *β* and *γ* are coefficients.

The results of the multiple-regression analysis for the four ground-anchor types are summarized in Table 1. The multiple correlation coefficients *R* for Type II and Type IV were notably high, whereas the coefficients for Type I (0.39) and Type III (0.43) were comparatively low. These lower coefficients may be attributable to the large variability in the evaluation peak frequencies for Type I and the narrow range of measured residual tensile forces for Type III. The coefficient of determination (R^2^), mean absolute error (MAE), root mean squared error (RMSE), and the ratios within ±5% and ±10% are also summarized in Table 1.

Figure 6b compares the actual tensile forces obtained from the lift-off tests with the tensile forces estimated using Equation (5) for the 144 ground anchors listed in Table 1 and installed on cut slopes along expressways, thereby providing a comprehensive assessment of the applicability of the proposed estimation method under practical field conditions. The ratio of the estimated tensile force, calculated from the evaluation peak frequency and surplus length, to the measured tensile force ranged from 89% to 120%, and the estimated tensile force was within ±5% of the measured value for 89% of the anchors and within ±10% for 95% of the anchors, demonstrating that the method appears to yield reliable results for the majority of anchors despite the inherent variability in site-specific conditions. However, further data spanning a wider tensile force range for each type of ground anchor will be needed to more rigorously validate the accuracy and applicability of the multiple-regression equations.

### 3.4. Acoustic-Barrier Supports

**Figure 7:**
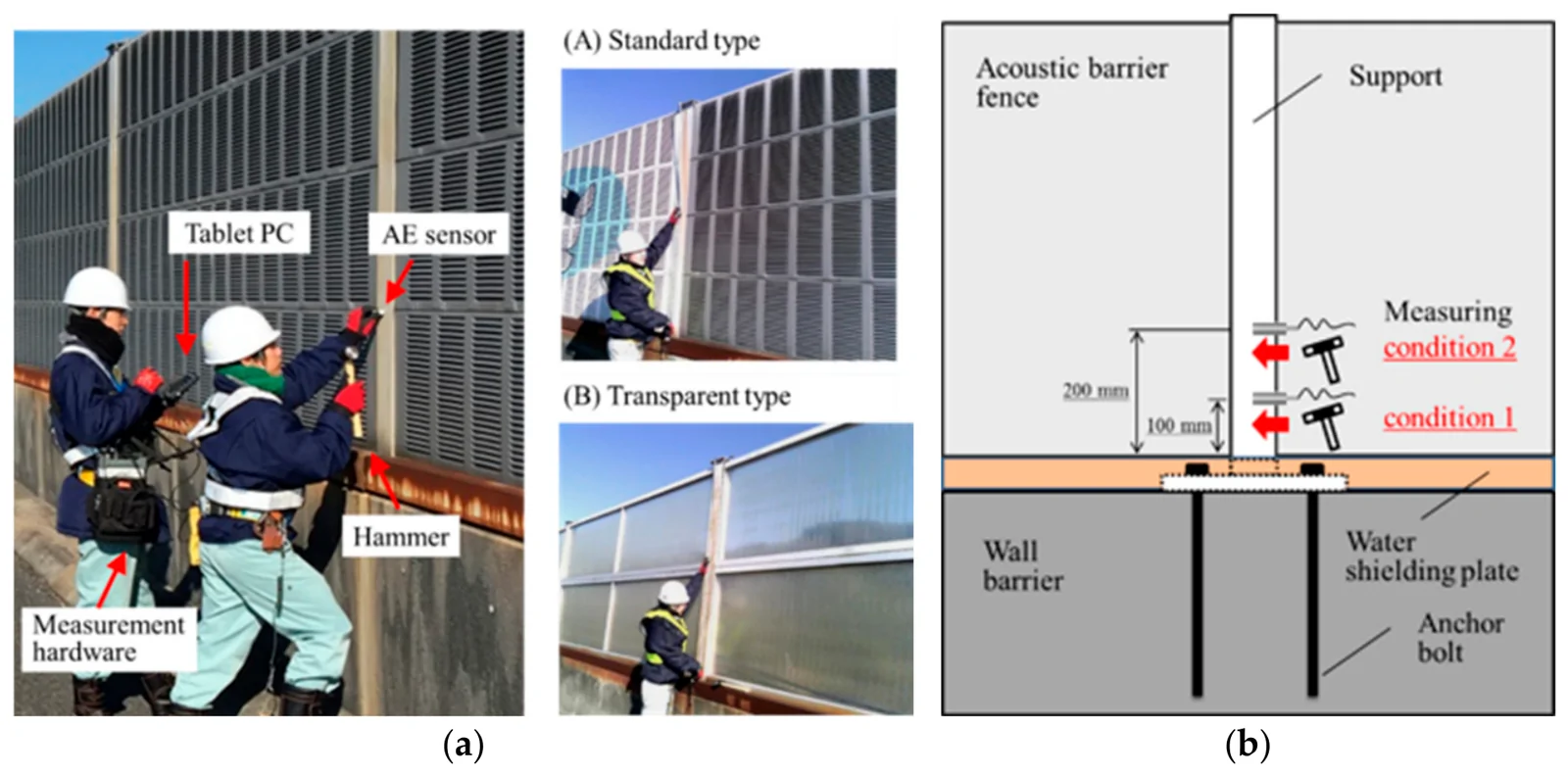
Field inspections of acoustic-barrier support. (**a**) Two types of acoustic-barrier support. (**b**) Inspection procedure: AE sensor positioned 100 mm and 200 mm above the water-shielding plate (Conditions 1 and 2), with hammering performed near the sensor.

Acoustic barriers installed along expressways to mitigate traffic noise are susceptible to loosening or detachment under wind pressure. To evaluate the practical applicability of the AE-based digital hammering inspection method, field measurements were conducted on acoustic-barrier support components. A total of 106 supports on barrier walls were inspected. As shown in Figure 7a, the supports comprised two types: a standard acoustic-barrier support and a transparent acoustic-barrier support, both approximately 2000 mm in height. The AE sensor was positioned 100 mm above the top of the water-shielding plate (measuring condition 1) and 200 mm above it (measuring condition 2), and hammering was performed in the vicinity of the sensor, as illustrated in Figure 7b.

**Figure 8:**
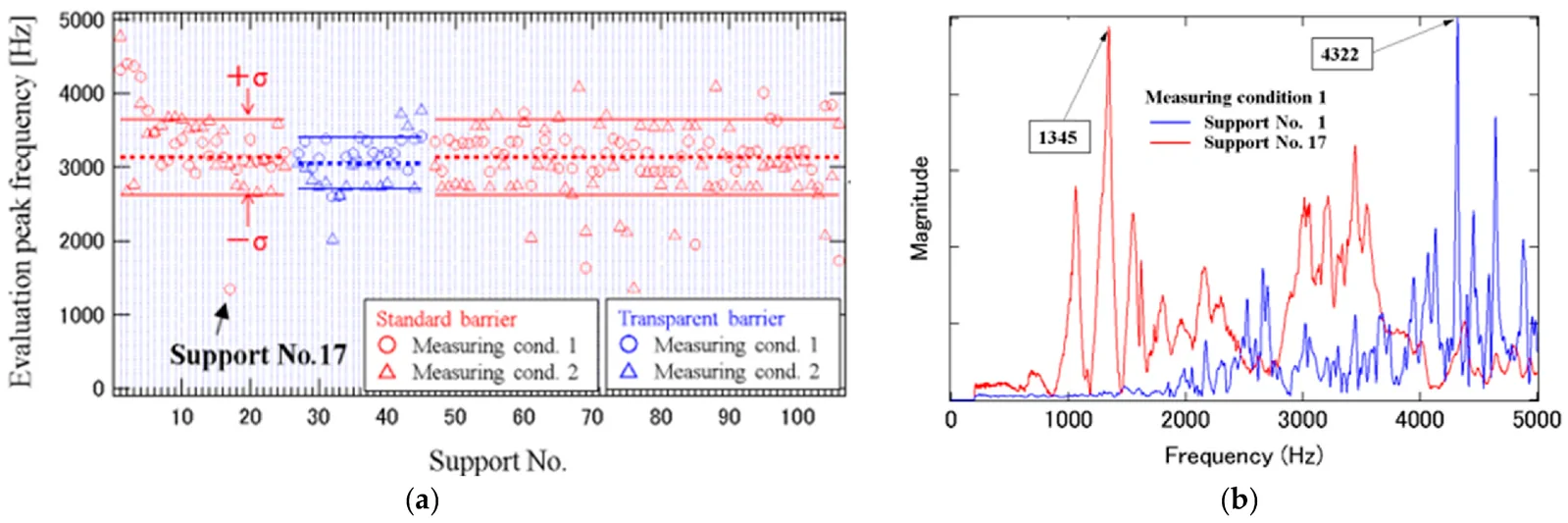
Field inspection results of acoustic-barrier support. (**a**) Inspection results of two types of support. (**b**) Comparison of frequency distributions for Support No. 01 and Support No. 17.

Figure 8a shows the evaluation peak frequencies obtained for the two types of supports. Data from the standard acoustic-barrier supports and the transparent acoustic-barrier supports are distinguished in red and blue, respectively. The average value (dotted line) and the upper and lower bounds corresponding to one standard deviation (solid lines) are also shown in Figure 8a. The difference in the mean evaluation peak frequency between the standard and transparent supports is approximately 100 Hz. There appears to be no significant influence attributable to the difference in sensor positions between measuring conditions 1 and 2.

Figure 8a shows that several acoustic-barrier supports exhibit evaluation peak frequencies markedly below the lower bound of one standard deviation. As an illustrative example, the frequency distributions for Support No. 01 and Support No. 17 are compared in Figure 8b. Support No. 17 exhibits an evaluation peak frequency of 1345 Hz, which is clearly lower than that of Support No. 01. This pronounced reduction suggests that the fixing force of Support No. 17 provided by its anchor bolts has diminished, resulting in the dominance of a lower-frequency vibration mode.

### 3.5. Guardrail Support and Embedded Length

**Figure 9:**
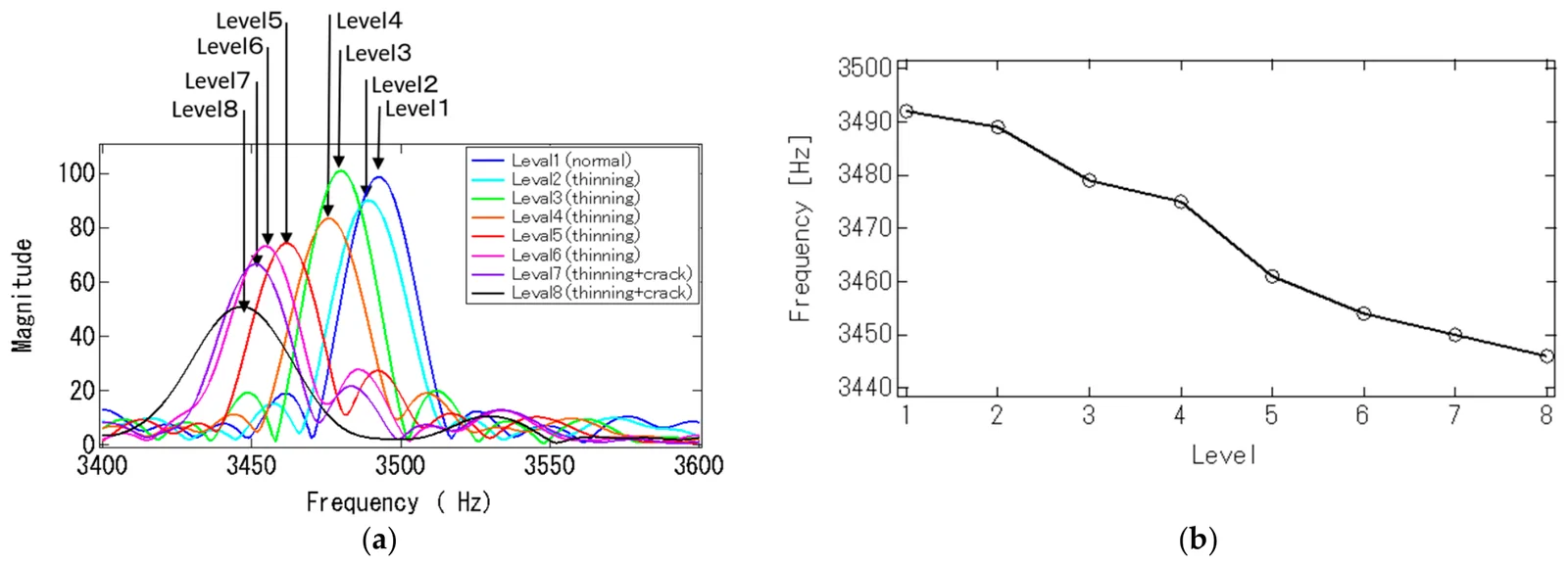
Inspection for simulated corrosion in guardrail supports. (**a**) Frequency distribution of the mock-up specimens. (**b**) Peak frequency change in the specimens.

Guardrail supports often undergo corrosion at ground level after extended service periods, particularly in regions where snow-melting agents are routinely applied, leading to progressive material loss and localized weakening that may ultimately compromise the load-bearing capacity of the support if not detected at an early stage. Because conventional inspection relies primarily on ultrasonic thickness measurements [19], which require point-by-point evaluation and can be time-consuming over long roadway sections, a more efficient screening technique is desirable for early detection of deterioration and for prioritizing supports that require detailed follow-up inspection. To this end, mock-up specimens with controlled, machined corrosion were prepared, and AE-based digital hammering inspection was applied to assess its feasibility as a rapid, non-destructive screening method, as shown in Figure 9a. To simulate corrosion at the ground line of a guardrail post, mechanical grinding was progressively applied around the entire circumference over a width of approximately 10 mm. Level 1 represents the normal (undamaged) condition. At Level 7, through-thickness penetration occurred over one-quarter of the circumference, whereas at Level 8, penetration occurred over one-half of the circumference. The machining allowed systematic variation in the remaining cross-section, enabling a clear examination of the relationship between structural degradation and vibration characteristics and providing a controlled environment for validating the sensitivity of the method. As illustrated in Figure 9b, the evaluation peak frequency exhibits a consistent downward shift as the simulated corrosion level increases, indicating that the reduction in stiffness associated with material loss can be sensitively captured by the proposed method. This trend suggests that the technique has sufficient resolution to detect even moderate levels of deterioration and may therefore serve as an effective preliminary screening tool prior to more detailed follow-up inspections in actual field applications.

**Figure 10:**
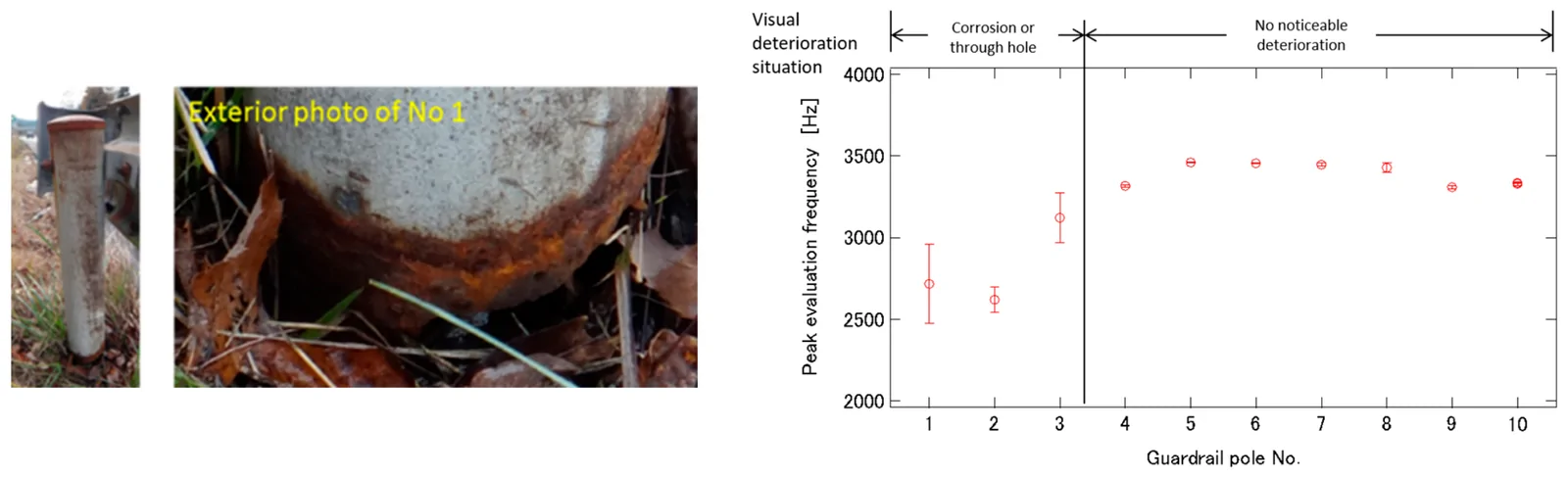
Field inspection results of corrosion damage in guardrail supports.

Field inspection results for ten guardrail supports are presented in Figure 10. Pronounced reductions in the evaluation peak frequency were observed for Supports No. 1 and No. 2, where substantial corrosion was also confirmed through visual examination. Since inspections were performed from four directions at 90° intervals around each support, some variability in the inspection results was observed for the same support when corrosion was present. The clear correspondence between the visually identified deterioration and the downward shift in peak frequency suggests that AE-based digital hammering inspection is sensitive to stiffness loss associated with corrosion-induced thinning. These findings indicate the potential of the method as an efficient screening technique for detecting deterioration in guardrail supports.

**Figure 11:**
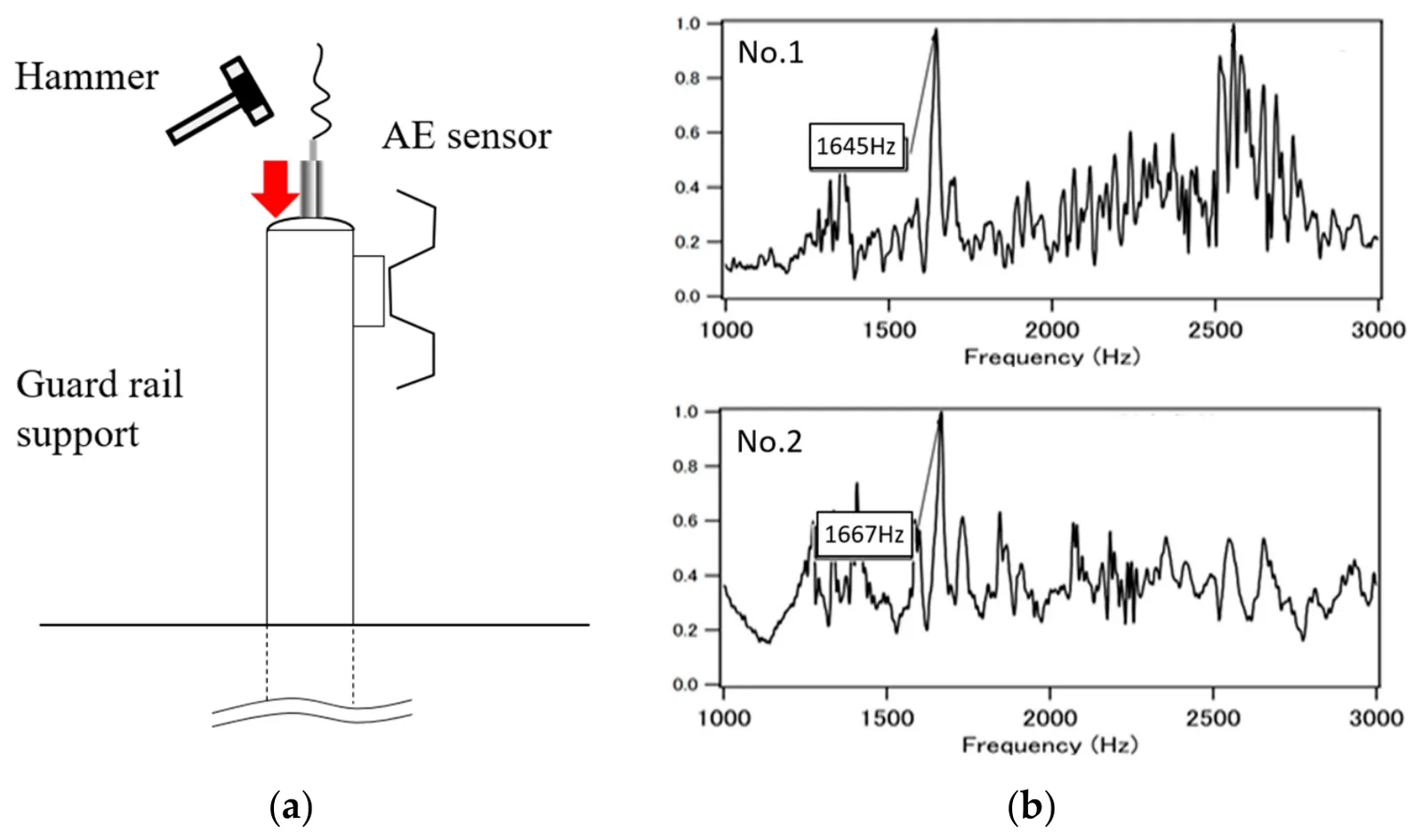
Field inspection of the embedded length of guardrail supports. (**a**) Inspection in their in situ configuration. (**b**) Frequency distributions.

Acquiring the embedded length of guardrail supports is essential for ensuring that the guardrail system maintains its intended safety performance, in addition to detecting corrosion at the ground level as described above. To evaluate this embedded length, the vibration response of each support is measured by pressing an AE sensor against the cap attached to the top of the support and striking the vicinity of the sensor, as illustrated in Figure 11a. The full length associated with the longitudinal vibration mode of a guardrail support is expressed by Equation (6) [20], which provides the basis for estimating the embedded portion from the measured natural frequency.

(6) $$f=\frac{\lambda }{2\pi L}\sqrt{\frac{E}{\rho }}$$

*f*: natural frequency, *λ*: constant of constraint condition of beam, *L*: vibration length [m], *E*: Young’s modulus [N/m^2^], *ρ*: density [kg/m^3^].

In this study, λ was set to π, corresponding to the first longitudinal vibration mode of a uniform rod.

Two Type-A guardrail supports (No. 1 and No. 2), each with an outer diameter of 139.8 mm, a wall thickness of 4.5 mm, a full design length of 2350 mm, and a design embedded length of 1650 mm, were scheduled for replacement due to pronounced corrosion at the ground line. Prior to their removal, on-site vibration measurements were conducted under actual service conditions, with the rail beams and steel caps still attached, as shown in Figure 11a. These measurements provided an opportunity to evaluate the applicability of AE-based digital hammering inspection to supports in their in situ configuration.

After extraction, the actual full lengths of the two supports were measured as 1620 mm. Given that the above-ground portion was 700 mm, the embedded depth was determined to be 920 mm. Because this value is substantially smaller than the design embedded depth of 1650 mm, it was confirmed that both supports had been installed with insufficient embedment, indicating the presence of unauthorized short guardrail supports.

Figure 11b presents the frequency distributions obtained from AE digital hammering inspection for the two supports. In both cases, a distinct evaluation peak frequency was observed, enabling a reliable estimation of the longitudinal vibration length. Table 2 summarizes the calculated full lengths, obtained by substituting the measured frequencies into Equation (6) using a Young’s modulus of 206 GPa and a density of 7900 kg/m^3^. The differences between the measured and estimated full lengths were on the order of several tens of millimeters for both supports. This level of agreement demonstrates that the AE-based digital hammering inspection method has practical potential as a screening technique for assessing the integrity of guardrail supports, particularly with respect to detecting insufficient embedded length.

The field test results indicate that AE-based digital hammering inspection may serve as a simple and effective means of evaluating both ground-level corrosion and the embedded length of guardrail supports.

### 3.6. Finger Joints

**Figure 12:**
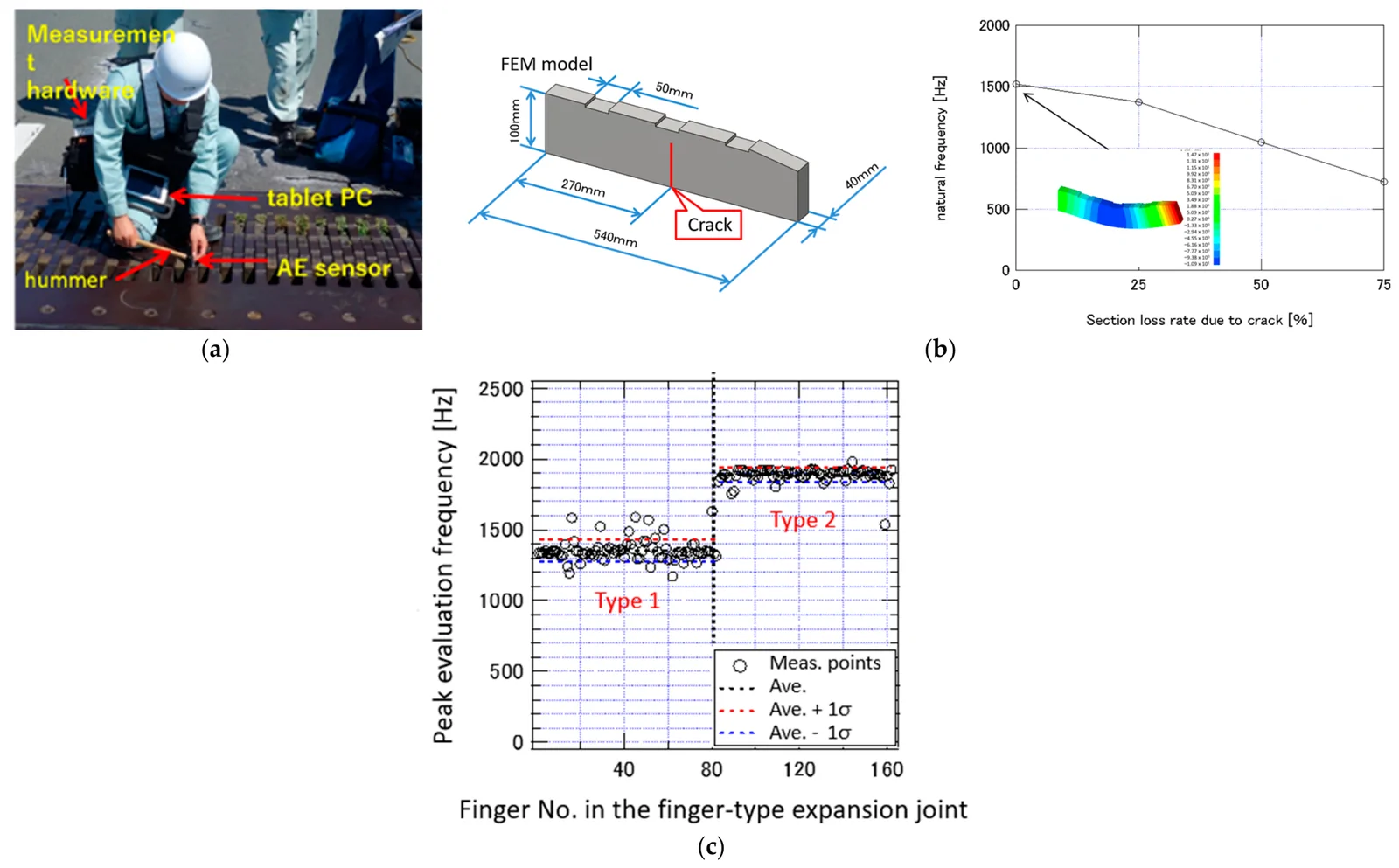
Inspection of finger joints subjected to fatigue damage. (**a**) Field inspection. (**b**) FEM analysis. (**c**) Field inspection results.

Finger joints (Figure 12a) are susceptible to fatigue cracking during service, and AE-based digital hammering inspection has been introduced as a rapid screening method for detecting such damage, particularly because conventional ultrasonic inspection requires comparatively long inspection times to identify fatigue cracks [21]. Figure 12b illustrates the FEM analytical model and corresponding analytical results for a 540 mm long finger portion of an expansion device. The model consists of 1 mm hexahedral solid elements, and four analytical cases were prepared by introducing a 1 mm wide crack at the center with cross-sectional deficiency rates of 0%, 25%, 50%, and 75%. Eigenvalue analysis was performed under a cantilever boundary condition, in which the finger root was fully constrained and the material properties of SS400 steel were applied (Young’s modulus: 206.0 GPa; density: 7900 kg/m^3^; Poisson’s ratio: 0.3). The evaluation peak frequency in the sound condition was 1521 Hz within the frequency range of interest, and it decreased progressively to approximately 750 Hz as the cross-sectional deficiency increased to 75%.

Figure 12c shows the field inspection results for finger joints Type 1 (540 × 100 × 40 mm) and Type 2 (750 × 100 × 40 mm) together with the mean values and standard deviation bands. Prior to AE digital hammering inspection, phased-array ultrasonic testing confirmed that no fatigue cracking was present. From Figure 12c, the obtained evaluation peak frequencies were around 1350 Hz for Type 1 and around 1900 Hz for Type 2. The discrepancy between the crack-free Type 1 FEM model and the field measurements is considered to be attributable to differences in modeling assumptions. The FEM analysis modeled only the finger joint body as a cantilever beam, whereas the field measurements included the influence of the root base plate connecting the joint to the supporting structure. Consequently, the measured natural frequencies were slightly lower than the analytical results. In addition, the standard deviation for each type was within 100 Hz, indicating stable measurement reproducibility. These results suggest that changes in the evaluation peak frequency can serve as an effective indicator for screening abnormalities in finger joints, including corrosion and structural deficiencies. While FEM simulations indicated that fatigue cracking may cause a shift in the evaluation peak frequency, verification of its correlation with actual crack occurrence in the field remains a subject for future work.

### 3.7. Concrete Bridge Deck Slab

Deterioration of concrete and corrosion of reinforcing bars are major factors that lead to the degradation of reinforced concrete structures [22,23]. These two processes often progress in a mutually related manner, and typical forms of damage include salt-induced deterioration and alkali–silica reaction (ASR). Such degradation mechanisms gradually weaken the structural capacity of concrete members and, in severe cases, may ultimately result in structural failure. Although close visual inspection and conventional hammering inspection have traditionally been employed to assess these conditions, the increasing demand for more quantitative and objective diagnostic methods has highlighted their limitations. In response, AE-based digital hammering inspection has been applied to concrete structures as a promising candidate for quantitative evaluation of deterioration, offering the potential to detect internal defects and material degradation with greater consistency and reliability.

**Figure 13:**
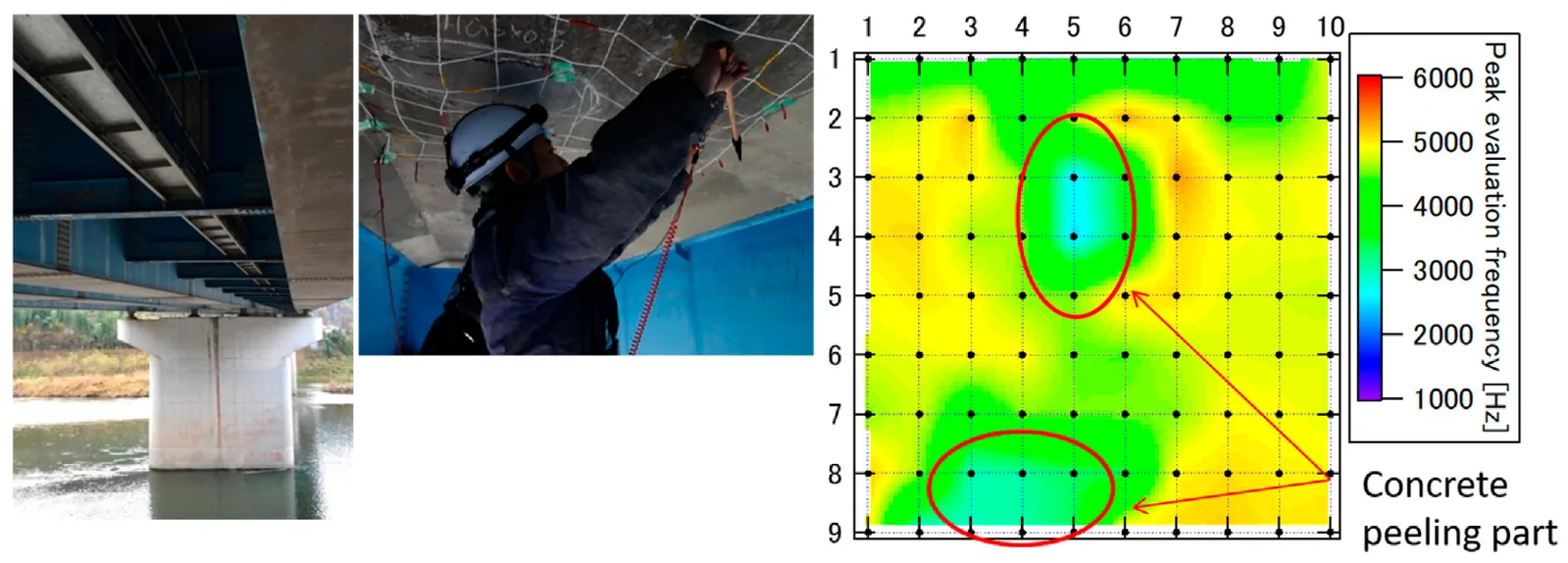
Field inspection of a concrete bridge deck slab.

Figure 13 shows the back side of a concrete bridge deck slab, the grid of inspection points, and the color-contour map of the evaluation peak frequency. Two areas highlighted with red circles correspond to locations previously judged as spalling based on a conventional hammering inspection conducted in advance. In these areas, the evaluation peak frequencies were in the range of 2–3 kHz, which is markedly lower than the average peak frequency observed across the inspected region, suggesting the influence of the low-frequency component generated based on Equation (4). This pronounced reduction is consistent with the presence of material degradation and suggests that the digital AE hammering inspection may be capable of capturing anomalies similar to those identified by the traditional method. These results further suggest that the digital hammering approach may offer diagnostic performance comparable to conventional hammering inspection, while also enabling quantitative frequency-based assessment.

### 3.8. Concrete Bridge Pier

The effectiveness of AE-based digital hammering inspection for evaluating the surface quality of a concrete bridge pier during the construction stage was also investigated, given that the surface condition plays a critical role in the subsequent deterioration of concrete, particularly in relation to freeze–thaw damage [24]. This examination aimed to determine whether early-stage surface irregularities or defects, which are known to accelerate freezing-induced degradation, can be quantitatively detected through variations in the measured peak frequency.

**Figure 14:**
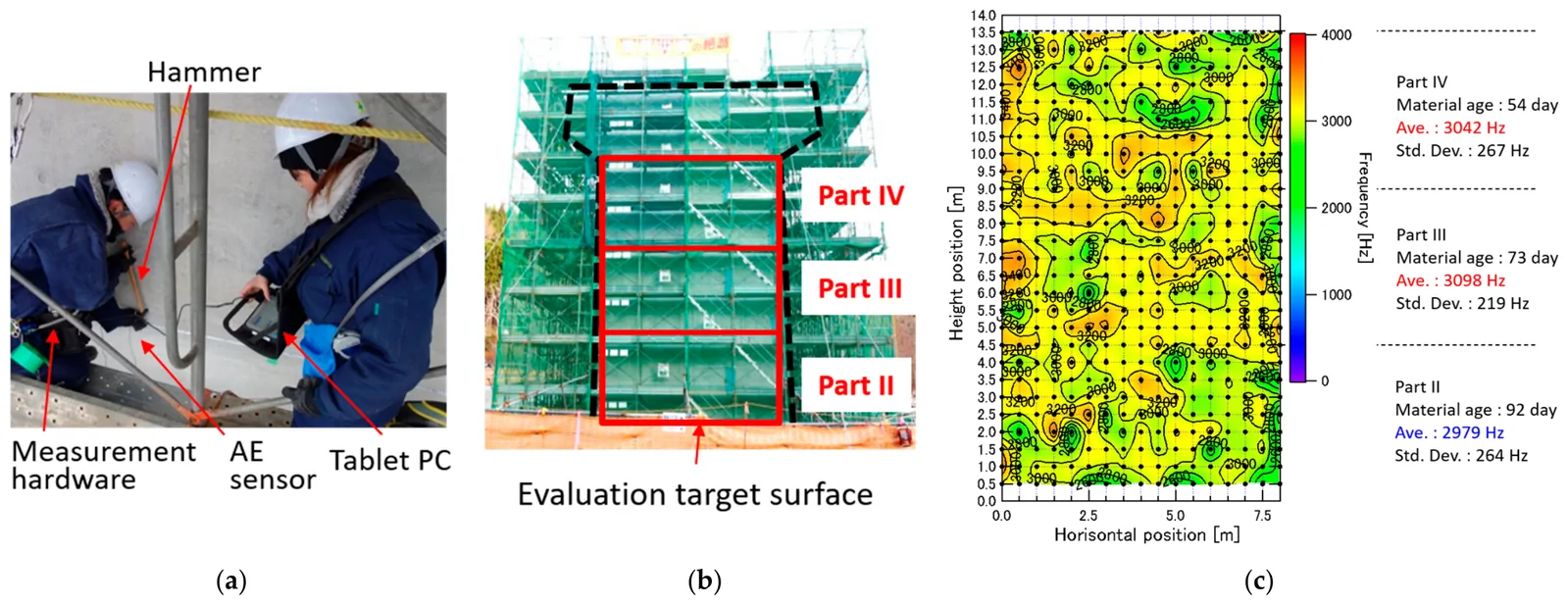
Field inspection of the quality of a concrete bridge pier. (**a**) Inspection scene. (**b**) Inspected bridge pier. (**c**) Color-contour map of the evaluated peak frequencies.

Figure 14 presents the inspection process (Figure 14a), the site appearance (Figure 14b), and the inspection results visualized as a color-contour map based on the evaluated peak frequency (Figure 14c). The concrete placement conditions are “regular” in the area of Part II and “carefully placed” in the areas of Part III and IV. The inspection was carried out at equal intervals of 50 cm lattice. The state of the concrete surface can be seen almost uniform without significant drop due to defects by the contour. On the other hand, the average evaluation peak frequencies in the carefully placed regions (Parts III and IV) were several tens to nearly 100 Hz higher than those in the regularly placed Part II. Considering the standard deviations of the inspection results in Parts II, III, and IV, the observed differences should be interpreted with caution. Nevertheless, a tendency toward higher evaluation peak frequencies was found in the carefully placed regions (Parts III and IV) compared with the regularly placed region (Part II). This tendency may be associated with increased stiffness due to the higher density and improved consolidation achieved through careful concrete placement.

### 3.9. Concrete Bridge Abutment

**Figure 15:**
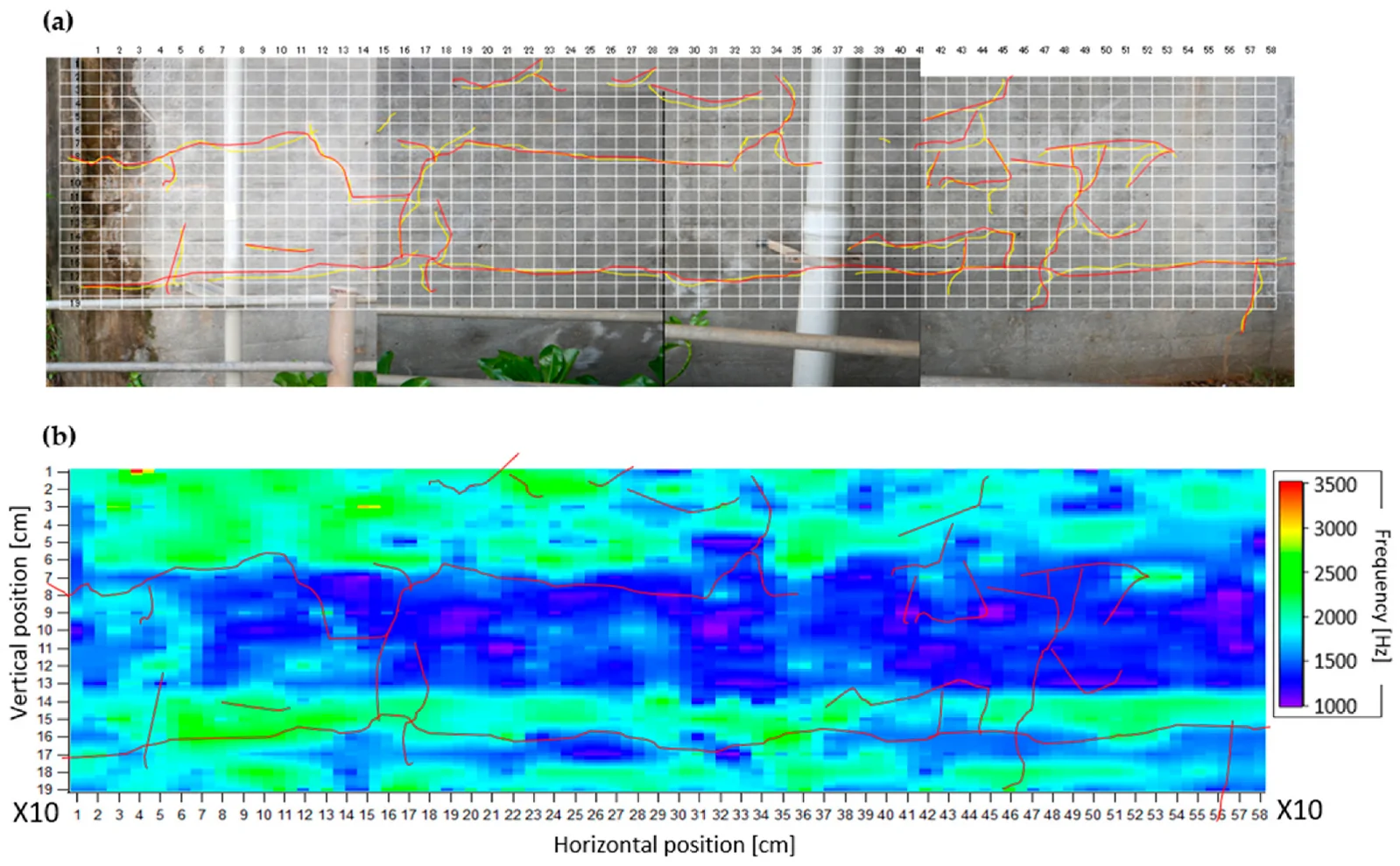
Field inspection of a concrete bridge abutment. (**a**) Photograph of the abutment and visible cracking indicated by red lines. (**b**) Results of inspection showing evaluation peak frequencies.

AE digital hammering inspection was conducted on a 1600 mm thick bridge abutment exhibiting concerns regarding ASR-induced deterioration. Core sampling at two locations revealed compressive strengths of 23.9 and 21.4 N/mm^2^, values notably lower than those typically observed in concrete bridge structures. In addition, long horizontal cracks and localized hexagonal cracking were identified on the abutment surface. The inspection covered an area of 1900 mm in the vertical direction and 5800 mm in the horizontal direction, with measurements taken on a 100 mm grid. Figure 15a shows a photograph of the abutment, the grid of inspection points, and the mapped locations of visible cracking indicated by red lines.

Figure 15b presents the results of the digital hammering inspection, which reveal lower evaluation peak frequencies along the long horizontal cracks and extremely low-frequency regions at localized points where ASR deterioration appears to be progressing. These reductions in frequency suggest a decrease in elastic modulus, consistent with the presence of ASR-related internal damage.

### 3.10. PC Grout Insufficient Filling

As an application of AE-based digital hammering inspection, the technique has been employed to identify insufficient grout filling within the sheaths of girders in pre-stressed concrete (PC) bridges. Inadequate grout filling can lead to corrosion of the pre-stressing wires housed in the sheath, posing a significant threat to the structural safety and long-term durability of the bridge. Because such corrosion often progresses invisibly within the sheath, early detection of grout-filling deficiencies is essential for preventing the sudden loss of prestress and subsequent structural degradation. Although X-ray transmission methods have been developed and used for this purpose, they remain time-consuming, require specialized equipment and traffic restrictions, and are therefore difficult to apply widely in routine inspections [25]. In contrast, AE-based digital hammering inspection offers a more practical and potentially scalable approach by enabling rapid, non-destructive, and quantitative assessment of grout-filling conditions based on frequency-response characteristics.

Twelve areas along the PC cables were selected for AE-based digital hammering inspection inside the box-shaped girder of a PC bridge. One of these areas had previously been identified, through drilling surveys, as exhibiting insufficient grout filling. In each area, measurements were taken at 10 positions located directly above the PC cable, together with additional measurements at vertical offsets of ±10 cm to obtain reference values representing sound conditions. The inspected regions had a concrete cover of approximately 55 mm over the PC cable and a web thickness of roughly 200 mm.

**Figure 16:**
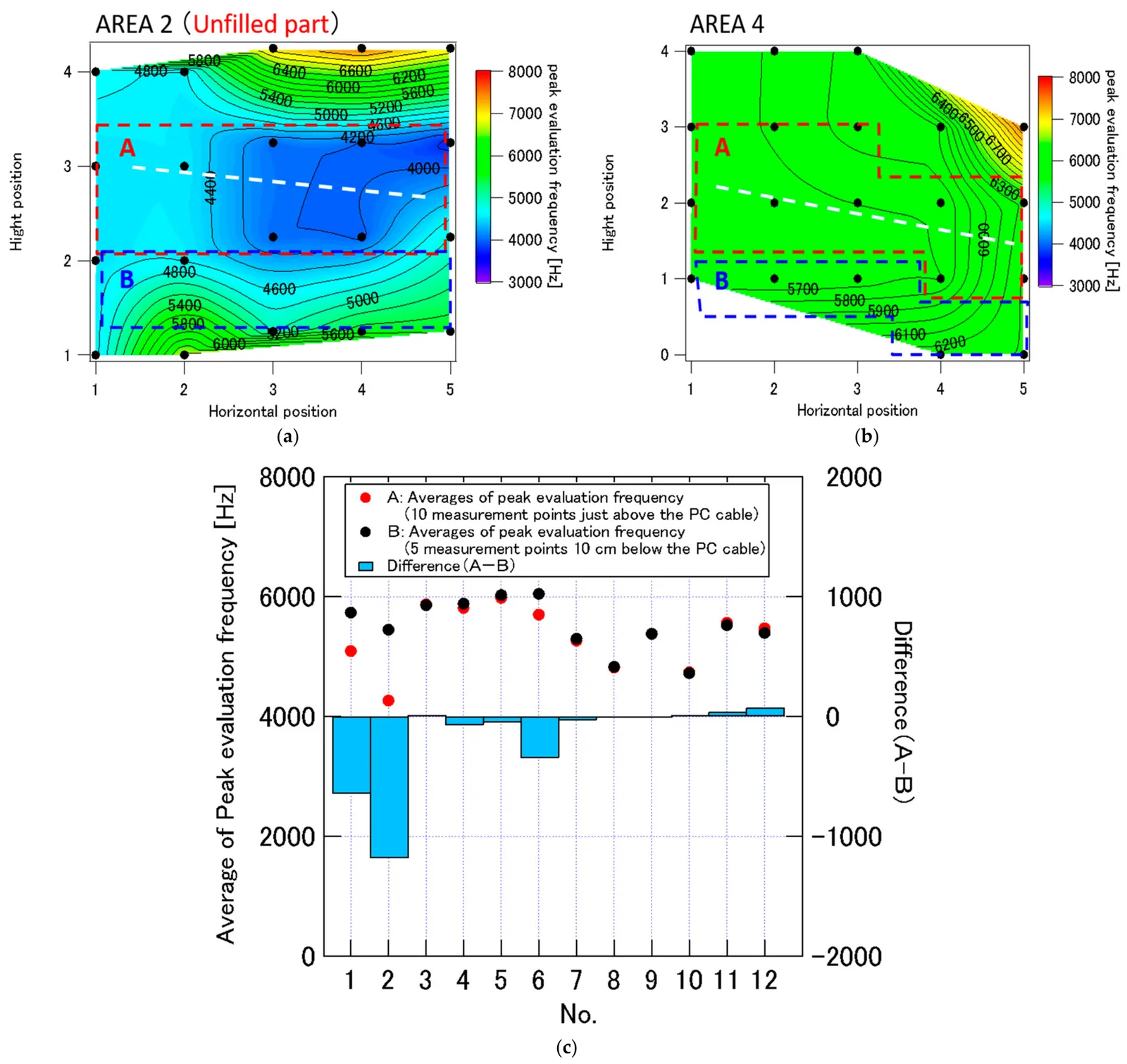
Field inspection of insufficient PC grout filling. (**a**) Color-contour map of the evaluation peak frequency for insufficient grout filling of the PC sheath. (**b**) Color-contour map of the evaluation peak frequency for sufficient grout filling of the PC sheath. (**c**) Field inspection results.

Figure 16a,b show color-contour maps of the evaluation peak frequency for AREA 2, where insufficient grout filling of the PC sheath had been confirmed, and AREA 4, where the grout filling was adequate. The position of the PC cable is indicated by white dotted lines. In AREA 2, the evaluation peak frequencies measured directly above the PC cable were markedly lower than those obtained at the vertical offset positions of ±10 cm, as well as those observed in the sound AREA 4. This clear contrast demonstrates that the grout-filling condition can be visually distinguished by comparing the frequency distribution immediately above the cable with that in the surrounding regions.

Figure 16c presents (A) the average evaluation peak frequency obtained from the ten measurement points located directly above the PC cable, (B) the average values from the five measurement points positioned 10 cm above and below the cable, and the corresponding difference (A–B) for all twelve inspected areas. The difference in evaluation peak frequency for AREA 2 is conspicuously larger than that of the other areas. This pronounced deviation is consistent with a localized reduction in stiffness associated with insufficient grout filling, suggesting the applicability of AE-based digital hammering inspection for the quantitative assessment of grout-filling deficiencies.

## 4. Conclusions

The AE-based digital hammering inspection system has been developed as an effective screening tool for the condition assessment of social infrastructure. The study clarified the fundamental principles governing digital hammering inspection by formulating the natural vibration characteristics of beam-shaped metal components and plate-shaped concrete structures in relation to their geometric dimensions and material properties. Field applications to post-installed expansion anchors, foundation bolts, ground anchors, acoustic-barrier supports, guardrail posts and their embedded lengths, finger joints, concrete bridge deck slabs, piers, abutments, and the grout-filling fraction of pre-stressed concrete members demonstrated the system’s broad applicability across diverse structural types. These findings confirm that the AE digital hammering inspection system provides a practical and versatile approach for diagnosing the integrity of social infrastructure and has strong potential to support both routine maintenance and long-term deterioration management.

## Figures and Tables

**Table 1:** Multiple-regression analysis of ground-anchor inspection results.

Anchor Type	Product No. of SE Corporation	Numbers	Anchor Head Diameter (mm)	Multiple Correlation Coef. R	Coef. α	Coef. β	Coef. γ	Coefficient of Determination R^2^	MAE (kN)	RMSE (kN)	Ratio of ±5%	Ratio of ±10%
Type I	F30TA	21	40	0.39	0.045	−0.446	201.3	0.15	14.2	17.7	0.52	0.71
Type II	F60UA	50	48	0.87	0.235	−5.413	296.3	0.76	9.4	11.7	0.92	0.98
Type III	F70UA	68	48	0.43	0.029	−0.196	397.0	0.19	3.6	4.5	1.0	1.0
Type IV	F40UA	5	40	0.72	0.033	−0.223	74.3	0.51	4.4	5.6	0.60	1.0
TOTAL	-	144	-	0.99	-	-	-	0.99	7.2	10.2	0.89	0.95

**Table 2:** Results for estimating the embedded length of guardrail supports in soil.

Support No.	Evaluation Peak Frequency [Hz]	Estimated Value (Full Length) [mm]	Measured Value (Full Length) [mm]	Difference Between Estimated and Measured [mm]
No. 1	1645	1552	1620	−68
No. 2	1667	1532	1620	−88

## Data Availability

Measurements and other relevant data are available from the corresponding authors upon request via email.
